# Resveratrol Couples Apoptosis with Autophagy in UVB-Irradiated HaCaT Cells

**DOI:** 10.1371/journal.pone.0080728

**Published:** 2013-11-19

**Authors:** Nicoletta Vitale, Annamaria Kisslinger, Simona Paladino, Claudio Procaccini, Giuseppe Matarese, Giovanna Maria Pierantoni, Francesco Paolo Mancini, Donatella Tramontano

**Affiliations:** 1 Department of Molecular Medicine and Medical Biotechnologies, University of Naples “Federico II”, Naples, Italy; 2 Institute of Oncology and Experimental Endocrinology, CNR, Naples, Italy; 3 Department of Medicine and Surgery, University of Salerno, Baronissi Campus, Salerno, Italy; 4 IRCCS MultiMedica, Milan, Italy; 5 Department of Sciences and Technologies, University of Sannio, Benevento, Italy; II Università di Napoli, Italy

## Abstract

UVB radiation causes about 90% of non-melanoma skin cancers by damaging DNA either directly or indirectly by increasing levels of reactive oxygen species (ROS). Skin, chronically exposed to both endogenous and environmental pro-oxidant agents, contains a well-organised system of chemical and enzymatic antioxidants. However, increased or prolonged free radical action can overwhelm ROS defence mechanisms, contributing to the development of cutaneous diseases. Thus, new strategies for skin protection comprise the use of food antioxidants to counteract oxidative stress. Resveratrol, a phytoalexin from grape, has gained a great interest for its ability to influence several biological mechanisms like redox balance, cell proliferation, signal transduction pathways, immune and inflammatory response. Therefore, the potential of resveratrol to modify skin cell response to UVB exposure could turn out to be a useful option to protect skin from sunlight-induced degenerative diseases. To investigate into this matter, HaCaT cells, a largely used model for human skin keratinocytes, were treated with 25 or 100 µM resveratrol for 2 and 24 hours prior to UVB irradiation (10 to 100 mJ/cm^2^). Cell viability and molecular markers of proliferation, oxidative stress, apoptosis, and autophagy were analyzed. In HaCaT cells resveratrol pretreatment: reduces UVB-induced ROS formation, enhances the detrimental effect of UVB on HaCaT cell vitality, increases UVB-induced caspase 8, PARP cleavage, and induces autophagy. These findings suggest that resveratrol could exert photochemopreventive effects by enhancing UVB-induced apoptosis and by inducing autophagy, thus reducing the odds that damaged cells could escape programmed cell death and initiate malignant transformation.

## Introduction

Although skin cancer is one of the most preventable cancer types by simply avoiding UV exposure, its incidence is on the rise, also because of life style changes [1]. In fact, prolonged skin exposure to pro-oxidant agents, such as UVB, can overwhelm built-in anti-oxidant defence, ultimately contributing to skin carcinogenesis [2]. Skin cells can respond to UVB-induced damage either by tolerating it, or repairing it via the activation of antioxidants and DNA repair mechanisms or, ultimately, undergoing programmed cell death when damage is massive. However, some damaged cells escape apoptosis, lose mitotic and differentiation control and finally become cancerous cells. Thus, induction of apoptosis and/or other death mechanisms, such as irreversible cell cycle arrest or autophagy, represent a key defensive strategy to ensure the removal of damaged and potentially carcinogenic cells [3]. UV-induced cell death involves distinct pathways, such as DNA damage, death receptor activation and formation of reactive oxygen species (ROS); these mechanisms are not mutually exclusive, but contribute to the overall UV-induced apoptosis [4], [5]. Therefore, the ideal chemopreventive agent for skin cancer should be able, not only to prevent UVB-induced cell damages and eliminate potentially carcinogenic damaged cells, but also to interfere with different signaling pathways.

Nutritional factors are estimated to contribute to preventing 20–60% of cancers around the globe [6], [7]. In particular, food antioxidants, such as resveratrol, (-)-epigallocatechin 3-gallate, genestein, beta-carotene, and lycopene, have attracted much interest because of their potential use in new preventive, protective, and therapeutic strategies for chronic degenerative diseases including skin cancer [8]–[12].

As for resveratrol, since the pioneer work of Jang in 1997 [13], data on animal studies and *in vitro* cell system, suggests that resveratrol protects skin from UV-induced photo-damaging and photo-aging. However, despite large efforts, the mechanisms underlying its chemopreventive effects remain still largely elusive [14], [15] mostly because the pleiotropic effects of resveratrol differ upon experimental systems, dose, concentration, and length of treatment.

For example, resveratrol differentially affects UV-induced death/pro-survival pathways in normal and tumor cells, may enhance/decrease UV-induced damages, and can interfere with UVA- and UVB-affected molecular events in skin keratinocytes independently of the redox balance [16]–[20]. In that, the dogma that the effects of resveratrol have to be ascribed solely to its antioxidant activity has been challenged by several reports showing that high concentrations of resveratrol can potently induce ROS production. [21]–[23].

The level of complexity of resveratrol effects is now increased by the possible implication of resveratrol in the regulation of autophagy [24]. In mammalian cells, autophagy occurs at low constitutive levels, to prevent the accumulation of damaged and malfunctioning cell components; this basal level is enhanced during starvation to provide an alternative source of energy. Autophagy principally serves as an adaptive mechanism of ‘programmed cell survival’, although, paradoxically, the self-digestive pro-survival functions of autophagy may lead to cell death, if carried on beyond a certain limit (type II programmed cell death) [25]–[27].

This complex and contrasting effects and the few and inconclusive human study warrant the uncertainties about the possible use of resveratrol in the clinic and call for more studies. Therefore, the commercialization of all sort resveratrol-containing products is alarming.

In the present work we have studied the combined effect of resveratrol and UVB on HaCaT cells, a well-known *in vitro* model of keratinocytes. In particular, we have tested the effect of acute (2 hours) or prolonged (24 hours) pretreatment with 25 and 100 µM prior to the irradiation of HaCaT cells with high and low doses of UVB. Our results demonstrate that 24-hour resveratrol pretreatment of HaCaT cells: **a**- reduces UVB-induced ROS formation; **b**-enhances UVB-induced apoptosis by increasing UVB-induced caspase 8 and PARP cleavage; **c**- induces autophagy per se and in irradiated cells. Hence, enhancement of UVB-induced apoptosis and induction of autophagy aid in the elimination of all UVB-damaged cells and may be responsible for resveratrol chemopreventive effect.

## Materials and methods

### 1. Materials

HaCaT cells, an immortalized, non-tumorigenic human keratinocyte cell line, were obtained from ATCC (LGC Standards SRL, Sesto San Giovanni, Milan Italy). Dulbecco Modified Eagle's Medium (DMEM), penicillin, streptomycin, foetal bovine serum (FBS), phosphate-buffered saline (PBS), and TripLE-express were purchased from Invitrogen S.r.l. (San Giuliano Milanese, Italy). Bovine serum albumin, sodium orthovanadate, sodium fluoride, sodium dodecyl sulfate (SDS), ammonium chloride (catalog # A0171), and leupeptin (catalog # L2884), were purchased from Sigma (Milano, Italy). Protease inhibitor cocktail tablets were purchased from Roche Diagnostics (Meylan, France). PVDF membranes and pre-stained protein standards were purchased from Invitrogen, S.r.l. (San Giuliano Milanese, Italy). Antibodies were purchased from the following suppliers: polyclonal anti-phospho-p44/42 MAP kinase (Thr202/Tyr204), polyclonal anti-phospho-Akt (Thr308), monoclonal anti-caspase-8 (1C-12), polyclonal anti-phoshpo-p38 Map-kinase (Thr180/Tyr182), polyclonal anti-phospho-p53 (Ser-15), polyclonal anti-LC3A/B, and anti-phoshpo-p70S6 kinase (Thr 389) antibodies (Cell Signaling Technology catalog # 9101, 9275, 9746, 9211, 2971, 9284, 4108, 9205, respectively) from Celbio S.p.A. (Pero, Italy); anti-phospho-Histone H2A.X (Ser139) monoclonal antibody, clone JBW301(catalog # 05-636) fromMillipore (Merck Millipore, Billerica, Massachusetts USA); polyclonal anti-p21(C-19), polyclonal anti-phospho S6 ribosomal protein (Ser240/244), monoclonal anti-Bcl-2 (100), polyclonal anti-Bax (P-19), *polyclonal anti-Beclin1 BECN1(H-300)* (catalog # sc-397, 2215, 509, 526, 11427 respectively) from Santa Cruz Biotechnology DBA (Milan Italy); monoclonal anti-p-27 antibody (kip-1, catalog # 610241) from BD Bioscience (Franklin Lakes, NJ USA); monoclonal anti-GAPDH antibody (catalog # G098, G041, respectively) from ABM Materials (Richmond, BC, Canada); anti-rabbit IgG and anti-mouse IgG, from Amersham Pharmacia (Buckinghamshire, UK); ECL System was purchased from Amersham Pharmacia (Buckinghamshire, UK). Bio-Rad assay and pre-stained protein standards were purchased from Bio-Rad (München, Germany). Pan caspases inhibitor Z-VAD (catalog # G7231 was purchased from Promega Italia (Milan, Italy).

### 2. Cell culture and cell proliferation assay

HaCaT cells, an immortalized, non-tumorigenic human keratinocyte cell line, were maintained in DMEM supplemented with 10% FBS and 1% antibiotics at standard conditions (37°C, 5% CO_2_, in a humidified incubator). For proliferation studies, HaCaT cells were seeded in 60 mm culture dishes in standard medium or in the presence of different concentrations of resveratrol (25 and 100 µM). At appropriate intervals, triplicate dishes were trypsinized and cell number was determined by counting cell suspension in a Neubauer hemacytometer. The values reported represent the mean ± S.D. of three independent samples per each experimental point.

### 3. UVB irradiation

HaCaT cells were cultured to 80% confluence and then treated with 25 and 100 µM resveratrol for 2 and 24 hours. Pan caspase inhibitor Z-VAD (50 µM) was added to resveratrol treated and non-treated cells 2 hours prior to UVB irradiation. At time of irradiation cells were washed with PBS, covered with a thin layer of PBS, and irradiated with UVB (10 to 100 mJ/cm^2^) without plastic lid. After the addition of fresh medium, cells were further incubated for the indicated times. The irradiating source consists of three lamps (Philips Ultraviolet 8 TL 20W/01 RS lamps; Philips, Eindhoven, Netherlands) generating UVB light in the range of 290–320 nm with an emission peak at 312 nm. Intensity of UVB irradiation was measured using a phototherapy radiometer (International Light, Newburyport, MA).

### 4. Measurement of ROS

The formation of ROS was evaluated by means of the probe 2′,7′-dichlorofluorescin-diacetate (H_2_DCF-DA, Sigma) [28]. Briefly, 20×10^4^ HaCaT cells/well were seeded in 24-well plates. HaCaT cells were pretreated for 2, 8 and 24 hours with 25 or 100 µM resveratrol before UVB irradiation (10 to 100 mJ/cm^2^). Fluorometric determination of intracellular ROS was estimated by loading the cells with H_2_DCF-DA (10 µM) for 15 min at 37°C; cells were washed twice with PBS buffer and plates were placed in a fluorescent microplate reader (Perkin Elmer LS 55 Luminescence Spectrometer, Perkin-Elmer Ltd., Beaconsfield, England). Fluorescence was monitored using an excitation wavelength of 485 nm and an emission wavelength of 538 nm. Each experimental point was performed in triplicates.

### 5. Western blotting

Cells were grown to sub-confluence in standard medium, then treated with resveratrol and/or UVB as indicated. Treatment of HaCaT cells with NH_4_Cl (20 mM) and leupeptin (100 µM) was carried out for 24 hours in the presence or the absence of resveratrol prior to UVB irradiation (10 to 100 mJ/cm^2^). Cells were harvested in lysis buffer (50 mM HEPES, 150 mM NaCl, 1 mM EDTA, 1 mM EGTA, 10% glycerol, 1% Triton-X-100, 1 mM phenylmethylsulfonyl fluoride, protease inhibitor cocktail tablet, 0.5 mM sodium orthovanadate, 20 mM sodium pyrophosphate) 15′ or 4 hours and 30′ after irradiation as indicated. The lysates were incubated for 30 min on ice and supernatants were collected and centrifuged for 10 min at 14,000 g. Protein concentration was estimated by a modified Bradford assay and 25 or 50 µg/lane of total proteins were separated on SDS 10% acrylamide gels and transferred to PVDF membranes. Membranes were treated with a blocking buffer (25 mM Tris, pH 7.4, 200 mM NaCl, 0.5% Triton X-100) containing 5% non-fat powdered milk for 1 hour at room temperature. Incubation with the primary antibody was carried out overnight at 4°C. After serial washings, membranes were incubated with the horseradish peroxidase-conjugated secondary antibody (rabbit 1∶2000, mouse 1∶2000) for 1 hour at room temperature. Following further washings of the membranes, chemiluminescence was generated by an ECL system.

### 6. Fluorescence Microscopy

Cells were grown on coverslips, washed with PBS, fixed with 4% paraformaldehyde, quenched with 50 mM NH_4_Cl and permeabilized with 0.2% Triton X-100 for 7 min. Microtubules were stained by using an antibody against alpha-tubulin detected with FITC-conjugate secondary antibodies (Jackson ImmunoResearch Laboratories, Inc).

Lysotracker Probe and monodansylcadaverin were used to label lysosomes and autophagosomes, respectively. Briefly, cells grown on coverslips were incubated with Lysotracker Probe (Molecular Probes) for 1 h at 37°C before fixation. Cells grown on bottom-glass dishes were incubated with monodansylcadaverin 50 µM (Sigma, Milan, Italy) in PBS for 10 min at 37°C and imaged *in vivo* in PBS.

Images were collected using a laser scanning microscope (LSM 510 META, Carl Zeiss Microimaging, Inc.) equipped with a planapo 63× oil-immersion (NA 1.4) objective lens. All image processing was done using LSM 510 software.

### 7. Statistical Analysis

Each data point represents the mean ± S.D. of triplicate measurements per each experimental point. All data have been analyzed by Student's t test. P<0.05 was considered as statistically significant.

## Results

### 1. Resveratrol reduces UVB-induced ROS production

**Figure 1:**
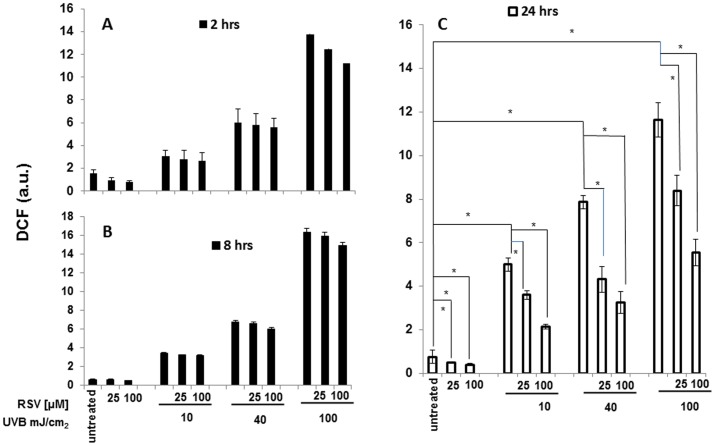
ROS evaluation in HaCaT cells exposed to resveratrol, UVB or both. HaCaT cells were pretreated for 2(panel A), 8 hours (panel B) or 24 hours (panel C) with resveratrol (25 and 100 µM) and then irradiated with UVB (10, 40, and 100 mJ/cm^2^). The levels of ROS were estimated indirectly by measuring the fluorescence emitted by dichlorofluoresceine (DCF) formed in proportion to the intracellular ROS 30′ after UVB irradiation. Graphs report mean values ± standard deviations of three independent measurement for each experimental point. RSV: resveratrol; a.u.: arbitrary units; * p<0.05.

Pretreatment of HaCaT cells with resveratrol for various length of time influenced UVB-induced ROS intracellular levels. Resveratrol was added at the concentration of 25 or 100 µM, 2, 8 or 24 hours prior to irradiation with 10, 40, or 100 mJ/cm^2^ UVB. UVB induced a strong dose-dependent increase of ROS production in HaCaT cells (Fig 1, panels A, B, C). 24-hours pretreatment with 25 or 100 µM resveratrol markedly reduced ROS levels in UVB-irradiated HaCaT cells. This effect was dependent on the dose of resveratrol and was present at each dose of UVB tested (Fig. 1, panel C). In particular, 25 µM resveratrol reduced by 32%, 46% and 28% ROS production induced by 10, 40 and 100 mJ/cm^2^ UVB irradiation, respectively, while 100 µM resveratrol reduced by 57%, 59% and 53% ROS production induced by the same irradiation intensities.

### 2. Resveratrol enhances UVB-induced death of HaCaT cells

**Figure 2:**
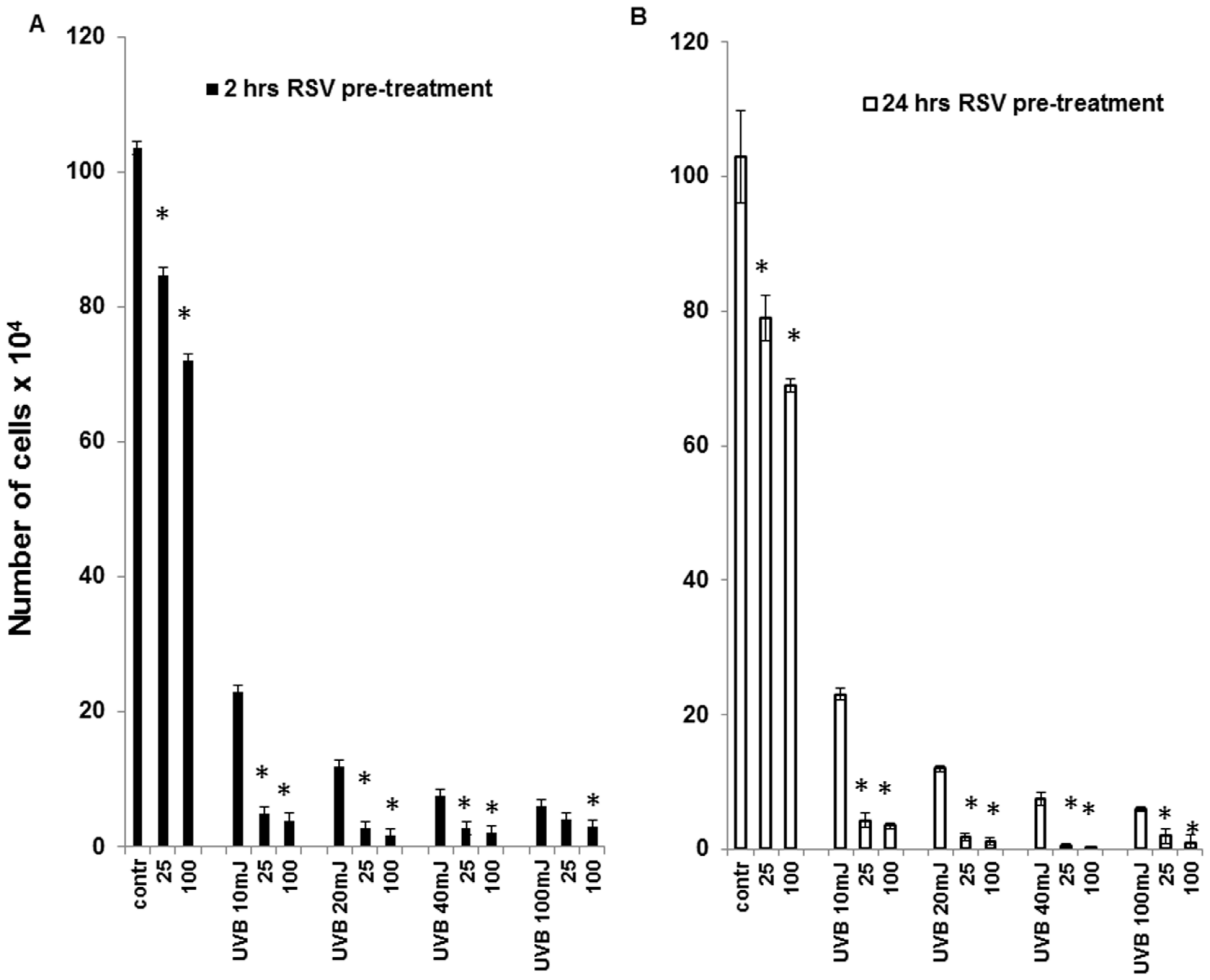
Resveratrol, UVB or both and HaCaT cells proliferation. Resveratrol (25 and 100 µM) was added to HaCaT cells for 2 hours (panel A) or 24 hours (panel B) and withdrawn prior to irradiation with UVB (10, 20, 40, and 100 mJ/cm^2^). Cell count was performed after 48 hours in culture in standard medium. Graphs report mean values ± standard deviation of three independent measurement for each experimental point. RSV: resveratrol; * p<0.05.

**Figure 3:**
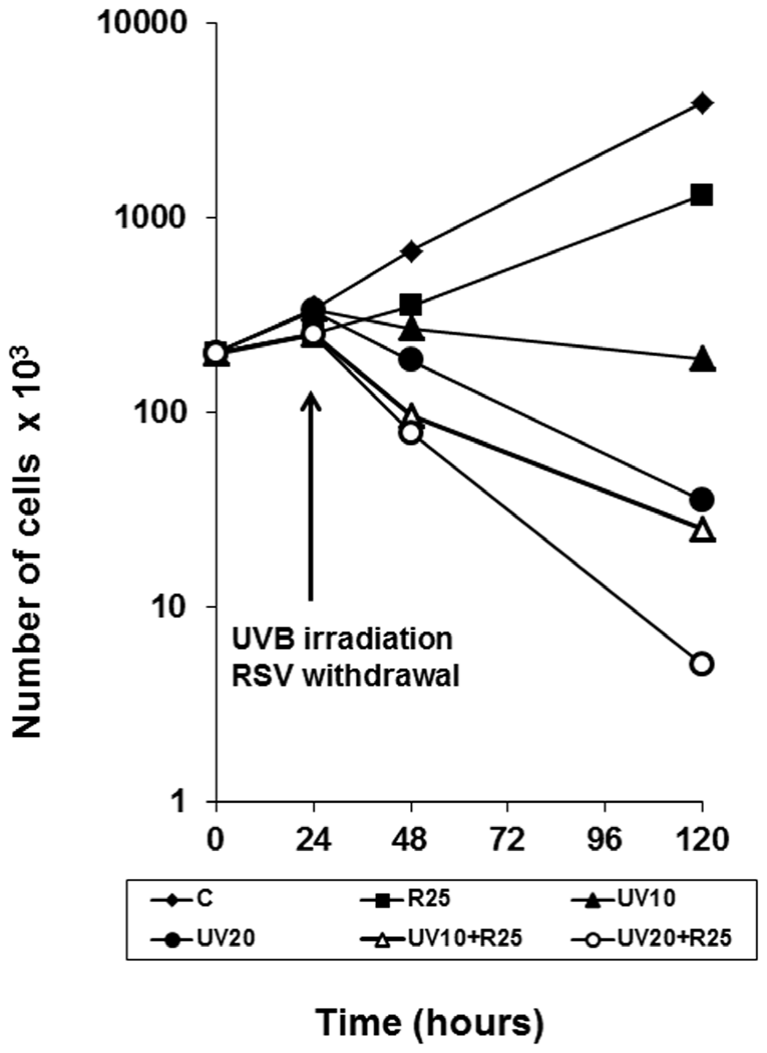
Growth curves of HaCaT cells exposed to resveratrol, UVB or both. Resveratrol (25 µM) was added to HaCaT cells for 24 hours and withdrawn prior to irradiation with UVB (10 or 20 mJ/cm^2^). Cells were counted after 48, and 120 hours in culture. **⧫** Untreated; ▪ RSV 25 µM; ▴UVB 10 mJ; Δ RSV + UVB 10 mJ; •UVB 20 mJ; o RSV + UVB 20 mJ.

UVB irradiation induces death of HaCaT cells in a dose-dependent fashion (Fig. 2, panels A, B). This effect parallels the UVB-induced ROS production showed in the previous figure. Therefore, we investigated whether the reduction of UVB-induced ROS increase caused by resveratrol pretreatment could influence the detrimental effect of UVB on HaCaT cells viability and proliferation. Cells were pretreated with 25 and 100 µM resveratrol for 2 and 24 hours before irradiation with different doses of UVB (10, 20, 40, and 100 mJ/cm^2^) and cell number was determined after additional 48 hours of culture in the absence of resveratrol. Pretreatment with 25 and 100 µM resveratrol for both 2 and 24 hours increased the detrimental effect of all doses of UVB tested (Fig. 2, panels A, B); therefore, the potentially protective effect of resveratrol, i.e. its ability to decrease UVB-induced ROS production, is not associated with increased viability of UVB-irradiated HaCaT cells. In addition, since we have previously reported that resveratrol-induced growth arrest in HaCaT cells is reversible, we have analyzed the long-term effect of 24-hour resveratrol pretreatment and UVB irradiation on HaCaT cells proliferation. As shown in Fig. 3, the detrimental effect of the conjoint treatment of UVB and resveratrol was observable also at low doses of UVB (10 and 20 mJ/cm^2^) and resveratrol (25 µM) and lasted up to 120 hours, regardless of the removal of resveratrol at time of irradiation.

### 3. Effect of H_2_O_2_ on ROS levels and proliferation of HaCaT cells

**Figure 4:**
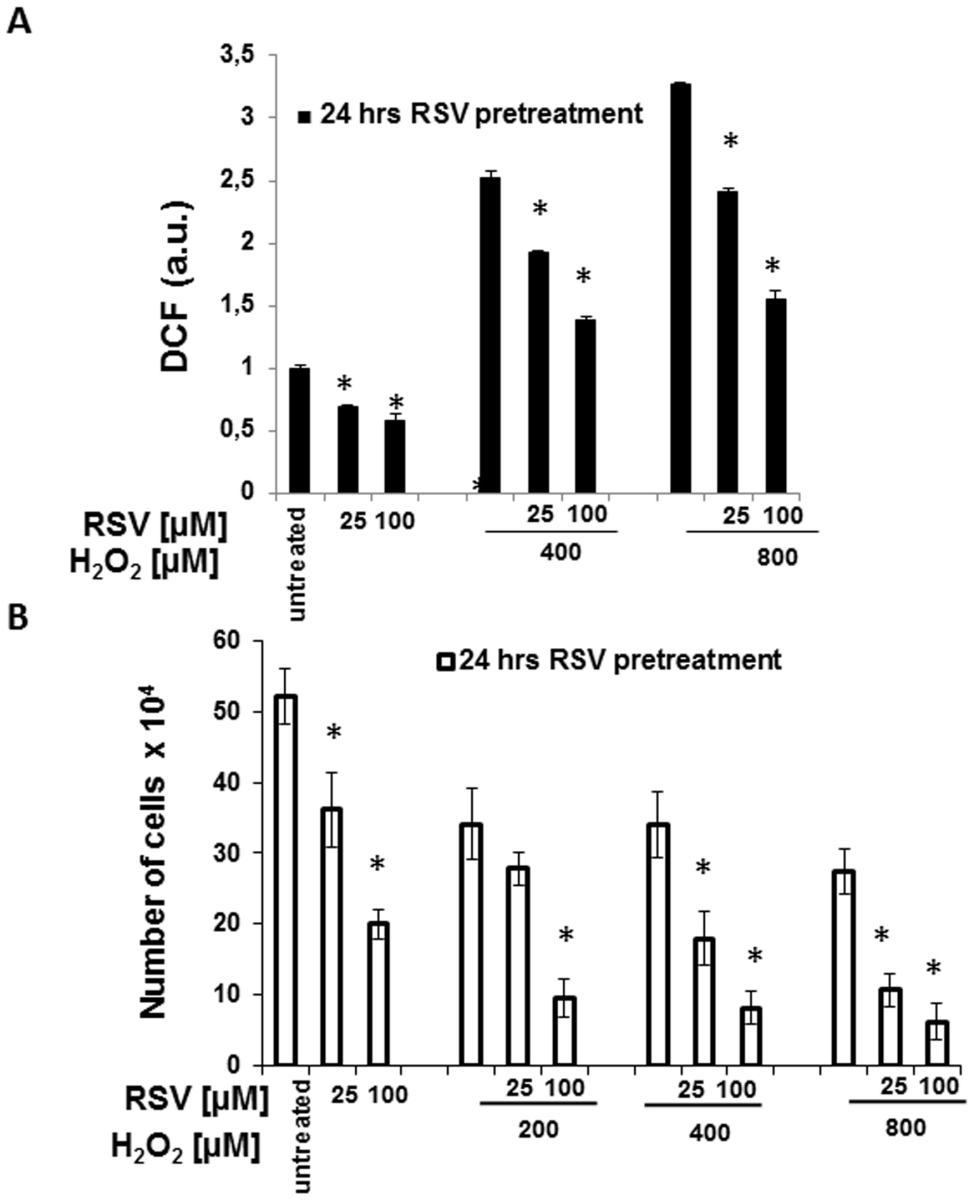
(A) ROS production in HaCaT cells and HaCaT cells were pretreated for 24 hours with resveratrol (25 and 100 µM) prior to the addition of H_2_O_2_ (200, 400, and 800 µM). The levels of ROS were estimated indirectly by measuring the fluorescence emitted by dichlorofluoresceine (DCF) formed in proportion to the intracellular ROS 30′ after addition of H_2_O_2_. Graphs report mean values ± standard deviation of three independent measurement for each experimental point. * p<0,05 **(B) HaCaT cell proliferation after exposure to resveratrol, H_2_O_2_ or both.** Resveratrol (25 and 100 µM) was added to HaCaT cells for 24 hours and withdrawn prior addition of H_2_O_2_ (200, 400, and 800 µM) and cell number was determined after additional 48 hours in culture. Graphs report mean values ± the standard deviation of three independent measurements for each experimental point. * p<0.05.

To provide more evidence about these apparently paradoxical effects of resveratrol on intracellular ROS levels and cell viability, we treated HaCaT cells with hydrogen peroxide, another inducer of ROS production. As shown in Fig. 4 (panels A), 400 and 800 µM H_2_O_2_ increased intracellular ROS, although at markedly lower levels than UVB. Also in this case, 24-hour resveratrol pretreatment reduced the increase in ROS level caused by H_2_O_2_ in a dose-dependent fashion. In particular, 25 µM resveratrol reduced by 24% and by 27% ROS production induced by 400 and 800 µM H_2_O_2_ respectively, while 100 µM resveratrol reduced by 46% and 53% ROS production in the same conditions. It is interesting that resveratrol reduced H_2_O_2_- and UVB-induced ROS production by the same percentage.

Analysis of cell proliferation in the same experimental conditions shows that H_2_O_2_ reduced HaCaT cell number and, after 24-hour pretreatment, both the lower and the higher doses of resveratrol (25 and 100 µM) caused a further marked decrease of cell proliferation at all the tested concentration of H_2_O_2_ (Fig. 4, panel B).

### 4. Early molecular events of the combined action of resveratrol pretreatment and UVB irradiation in HaCaT cells

**Figure 5:**
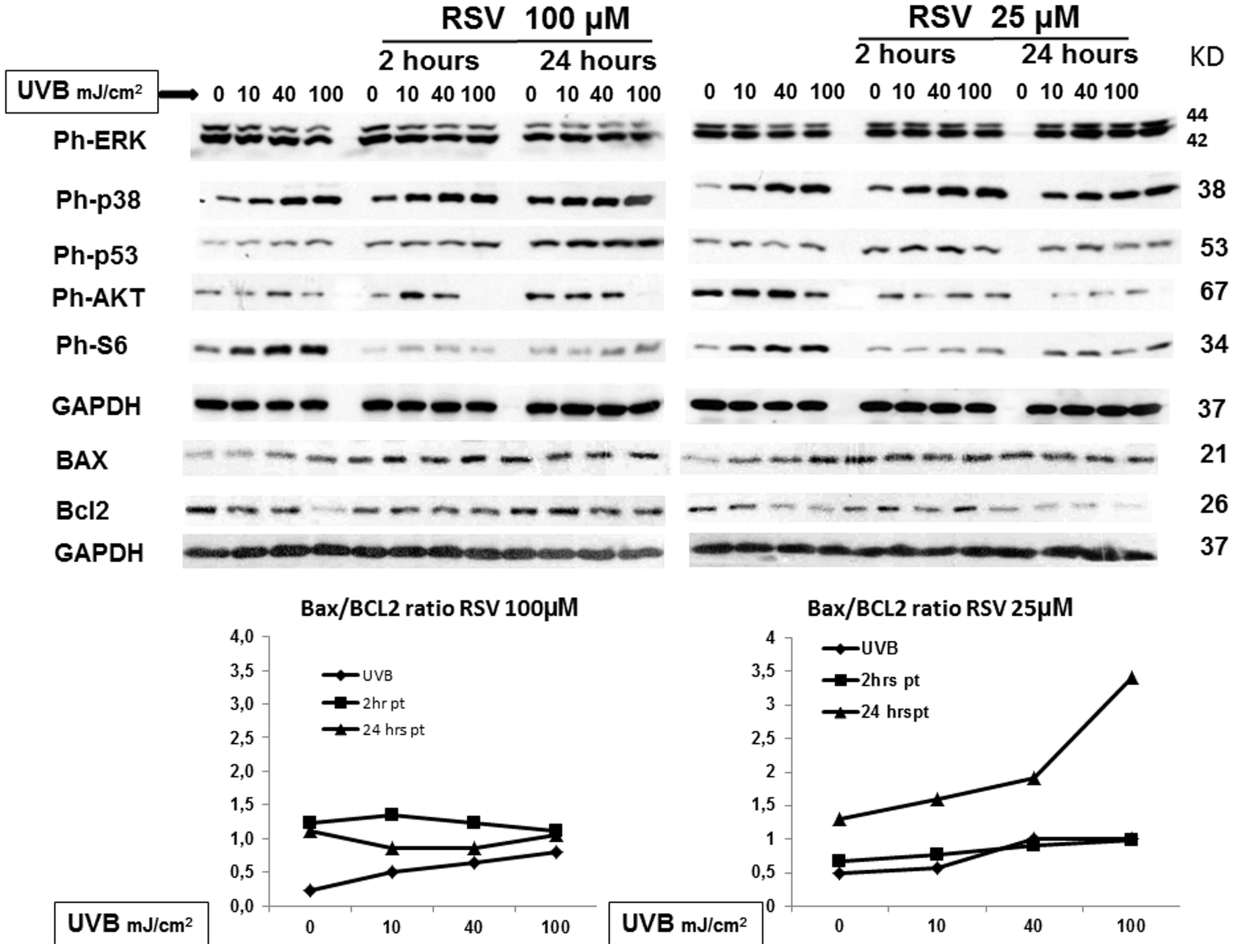
Multiple pathway analysis in HaCaT cells exposed to resveratrol, UVB or both: earlier time point (15 minutes). HaCaT cells were pretreated with 25 and 100 µM resveratrol for 2 and 24 hours prior irradiation with UVB (10, 40, and 100 mJ/cm^2^). Cell lysates were collected 15′ after UVB irradiation. Phosphorylated or not phosphorylated protein levels were analyzed by western blot. The figure shows representative blots analyzing the levels of the phosphorylated forms of ERK1/2, p38, p53, AKT, and S6, and the levels of BAX, and Bcl2.GAPDH was used as loading control.

To gain insight into the molecular mechanisms involved in the reduction of cell viability induced by the conjoint exposure of HaCaT cells to both UVB and resveratrol, we evaluated the expression levels of molecular targets known to be involved in the death/survival/proliferation pathways [29]–[34]. In particular, (Fig. 5) 15′ after UVB irradiation, 2 hour pretreatment with 100 µM resveratrol had not substantially effect on UVB-induced ERK phosphorylation, while 100 µM resveratrol substantially reduced ERK activation after 24 hour pretreatment. On the contrary, 25 µM resveratrol maintained ERK activation above UVB-induced level both at 2 and 24 hours resveratrol pretreatment. In the same experimental setting, both times of pretreatment and concentrations of resveratrol induced p38 and p53 activation above UVB-induced levels. Concomitantly, Bax/Bcl2 ratio substantially increased in cells pretreated for 24 hours with 25 µM resveratrol prior UVB irradiation. These results are all consistent with the hypothesis that resveratrol protective effect in response to UVB irradiation is exerted *via* early activation of well-known apoptosis triggers such as ERK, p38, p53, and increase of Bax/Bcl2 ratio. Furthermore, to investigate the effect of resveratrol pretreatment on survival pathways of irradiated HaCaT cells, we analyzed the activation of AKT and S6, the final read-out of the m-TOR pathway. At both concentrations and times of pretreatment, resveratrol reduced UVB-induced phosphorylation of the two proteins. Taken together these results indicate that resveratrol pretreatment also triggers traits of autophagy in response to UVB irradiation and suggest that this polyphenol is able to couple apoptosis and autophagy.

### 5. Resveratrol enhances UVB-induced Caspase 8 and PARP cleavage and regulates Beclin1 expression

**Figure 6:**
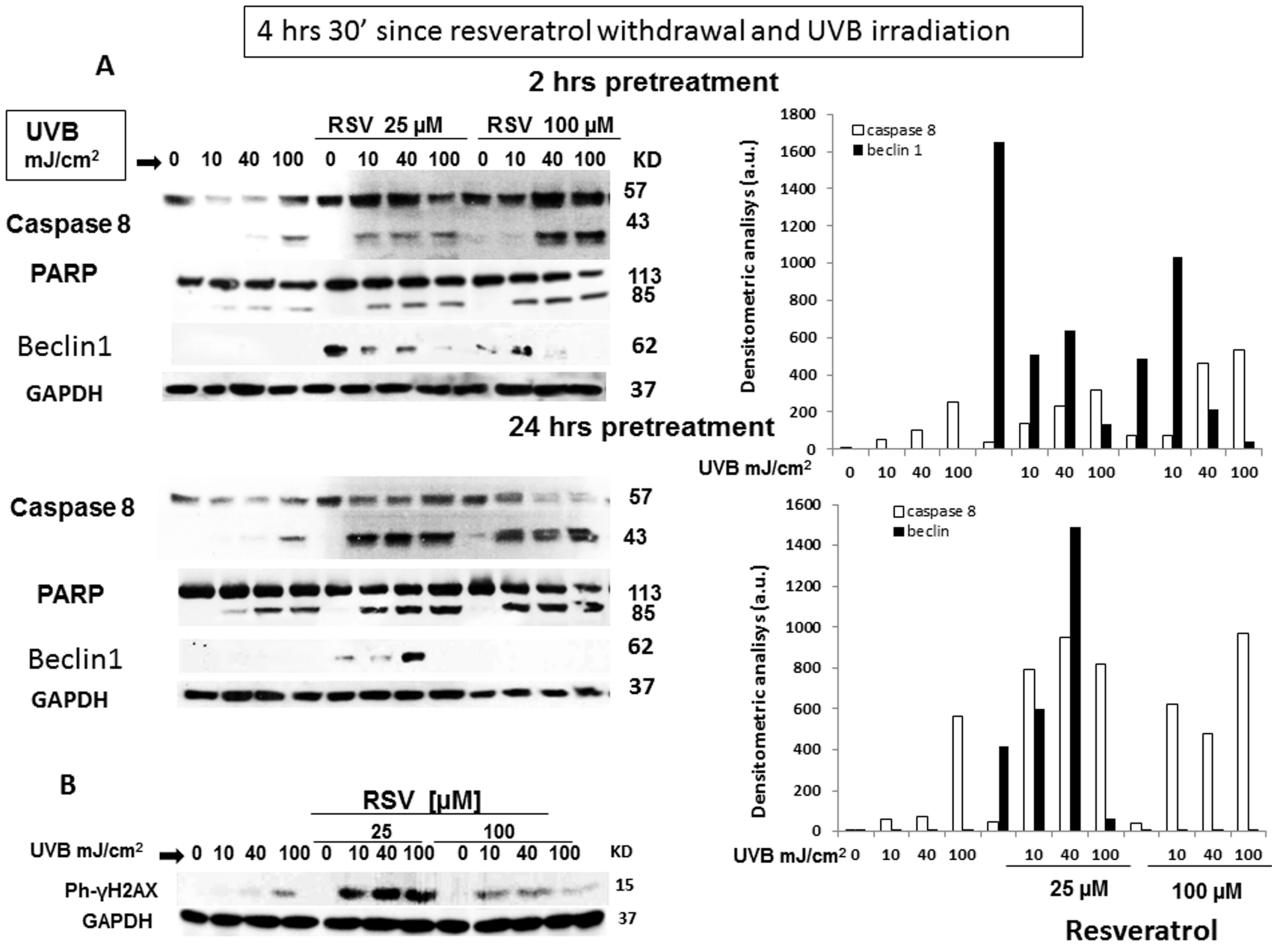
Cleavage of caspase 8 and PARP in HaCaT cells exposed to resveratrol, UVB or both. HaCaT cells were treated with resveratrol (25 and 100 µM, 2 and 24 hours), UVB (10, 40, and 100 mJ/cm^2^ alone or pretreated for 2 or 24 hours with resveratrol (25 and 100 µM) prior irradiation with UVB (10, 40, and 100 mJ/cm^2^). Lysates were collected 4 hours and 30′ after irradiation and resveratrol withdrawal and challenged with anti-caspase 8 and anti PARP antibodies recognizing the cleaved forms of the two proteins (panel A), or with anti-Beclin 1. Panel B reports the densitometric analysis of caspase 8 cleaved band and Beclin 1 at both 2 and 24 hours prior irradiation. The same lysates were challenged with anti γ-H2AX, recognizing the phosphorylated form of the histone (Panel D). GAPDH was used as loading control.

To further look into this possibility, we analyzed caspase and PARP cleavage as well as H2AX activation. In particular, caspase-8 is an initiator protease recruited to the death inducing signaling complex during apoptosis initiated by death receptors. UVB are able to activate caspase 8 cleavage in the absence of death receptor ligand, thus initiating the extrinsic apoptosis cascade [35]. On the other hand, PARP cleavage is a marker of the intrinsic apoptosis pathway. Therefore, caspase 8 and PARP were detected 4 hours and 30′ after UVB irradiation and resveratrol withdrawal in HaCaT cells pretreated with 25 and 100 µM resveratrol for 2 or 24 hours before UVB irradiation. As shown in Fig. 6 A, UVB increased caspase 8 and PARP cleavage in a dose-dependent fashion, and resveratrol pretreatment further enhanced these effects. It is of note that, in the same setting, resveratrol alone was not able to promote cleavage of either caspase 8 or PARP. Although we already obtained molecular evidence that resveratrol enhances also autophagy in UVB-exposed HaCaT ceIls (Fig. 5), we wanted to provide further indications on this matter analyzing a positive marker of autophagy that could allow a better evaluation of the respective contribution of autophagy and apoptosis to cell death in the present experimental setting. Therefore, we analyzed the expression of Beclin1(Atg6), which is an essential autophagy effector with an important role in the cross-talk with the apoptosis pathway. Although Beclin1 is enzymatically inactive, it governs the autophagic process by regulating PtdIns3KC3-dependent generation of phosphatidylinositol-3-phosphate (PtdIns(3)P) and the subsequent recruitment of additional Atg proteins that orchestrate autophagosome formation. As shown in Fig. 6, resveratrol regulates the expression of Beclin1 in a time- and dose-dependent fashion both alone or in combination with UVB irradiation. In that, at 2 hour pretreatment, resveratrol increased beclin1 expression at both 25 and 100 µM, while at 24 hours pre-treatment beclin 1 expression was observable only in cells pre-treated with 25 µM resveratrol. In addition, resveratrol-dependent beclin1 expression decreased at increasing doses of UVB, disappearing at 100 mJ/cm^2^ UVB. In the present experimental setting, beclin 1 expression inversely correlates with both caspase 8 and PARP cleavage. Thus, depending on time of pre-treatment/concentration of resveratrol and dose of UVB, both apoptosis and autophagy can be triggered.

H2AX is one of several genes coding for histone H2A that undergoes phosphorylation on Ser 139 (γ-H2AX) rapidly in response to DNA double-strand breaks induced by exogenous stimuli, such as ionizing radiation, and mediates the formation of clusters of proteins at the site of damage [36]. Apoptosis is tightly related to DNA damage and γ-H2AX is considered the most sensitive marker for detecting DNA damage. Pretreatment of HaCaT cells with 100 and 25 µM resveratrol prior to UVB irradiation potently increased γ-H2AX phosphorylation. Conversely, resveratrol alone had no effect on H2AX phosphorylation (Fig. 6 B). Taken together, these results strongly support the idea that, in HaCaT cells, resveratrol potentiates UVB-induced apoptosis, although it is not a pro-apoptotic stimulus by itself.

### 6. Late molecular events of the combined action of resveratrol pretreatment and UVB irradiation in HaCaT cells

**Figure 7:**
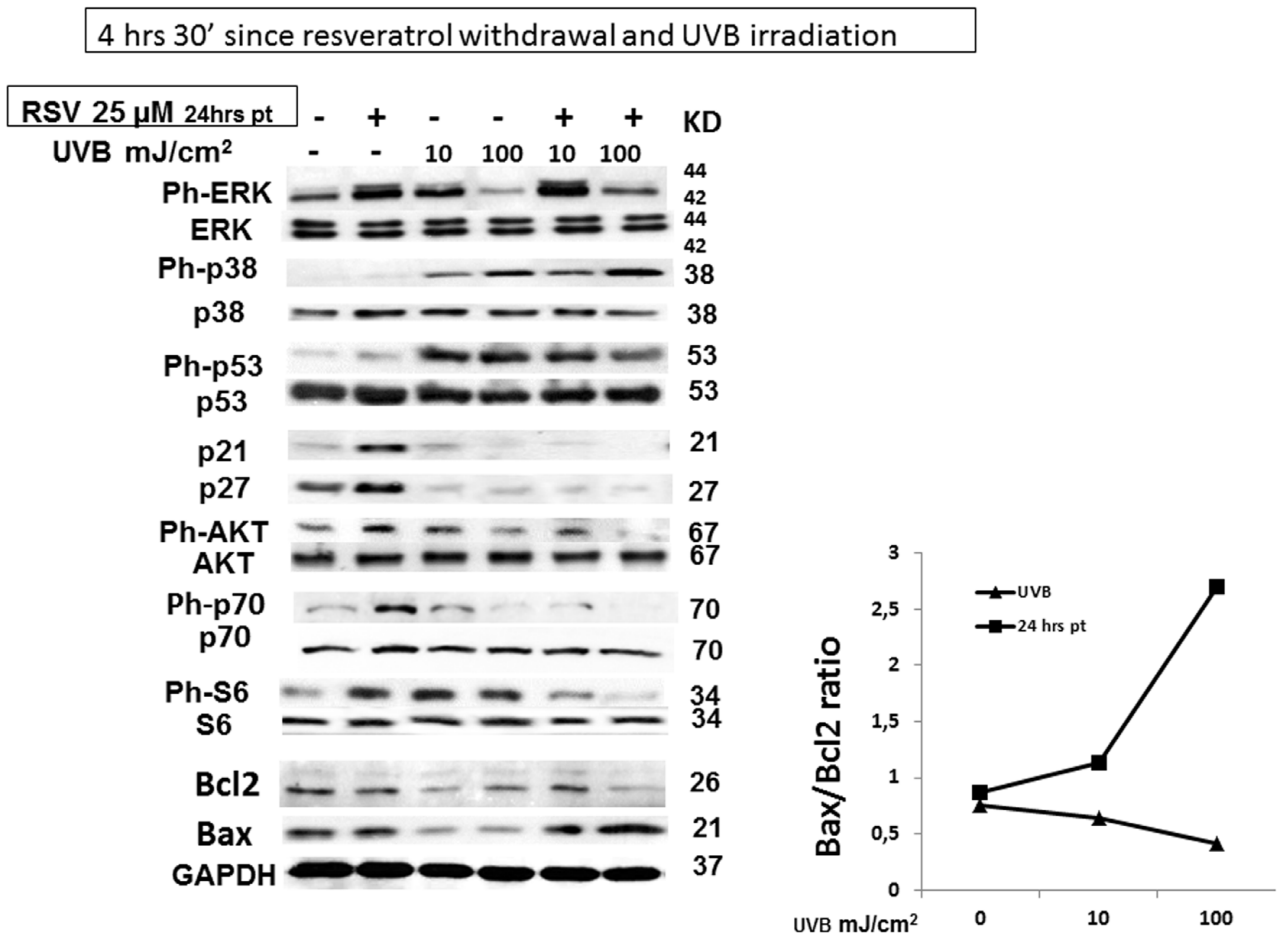
Multiple pathway analysis in HaCaT cells exposed to resveratrol, UVB: later time point (4.5 hours after resveratrol withdrawal and UVB irradiation). HaCaT cells were treated with 25 µM resveratrol for 24 hours prior to irradiation with UVB (10, and 100 mJ/cm^2^). Cell lysates were collected after 4 hours and 30′ resveratrol withdrawal and UVB irradiation. Phosphorylated or non-phosphorylated protein levels were analyzed by western blot. Total cell lysates (30 µg) were challenged with antibodies recognizing the phosphorylated forms of ERK1/2, p38, p53, AKT, p70, and S6 and or the native form of p21, and p27. Antibodies recognizing the non-phosphorylate form of ERK1/2, p38, p53, AKT, p70, S6 and GADPH were used as loading control.

Furthermore, we analyzed the expression or the activation of several markers of growth arrest, proliferation, and autophagy 4 hours and 30′ after resveratrol withdrawal and UVB irradiation, the same setting in which we observed caspase 8 and PARP cleavage and H2AX activation. Since, 24 hour-pretreatment with 25 µM resveratrol was sufficient to activate caspase 8 and PARP in UVB-irradiated HaCaT cells, we focused our attention on the effects of the lower concentration of resveratrol, which, in addition, is closer to the concentrations attainable in vivo with diet. As shown in Fig. 7, after 4 hours and 30′ ERK phosphorylation remained still increased in the resveratrol pretreated samples compared to resveratrol untreated cells both in non irradiated or UVB irradiated cells in accordance with recent findings showing that apoptosis is associated with sustained activation of ERK 1/2 [37], [38]. A similar behaviour was abserved for p38. Differently, a significant decrease in p53 phosphorylation was observed in resveratrol pretreated and UVB-irradiated HaCaT cells. It has been reported that resveratrol induces growth arrest through increase of p21 and p27 levels [39], on the other hand, UVB induced p21 and p27 degradation in HaCaT cells and resveratrol sustained this process in spite of its 4 hours and 30′ withdrawal, thus increasing apoptosis. This result is in line with those of Lei et al reporting that UVB-induced p21 degradation enhance UVB-induced apoptosis of premalignant keratinocytes with a p53 defect to eliminate damaged cells and therefore prevent skin cancer development [40]. Moreover, also Bax/Bcl2 ratios remained elevated.

Finally, although in non-irradiated HaCaT cells pretreated with resveratrol, phytoalexin withdrawal induced a significant increase in the phosphorylation of AKT, p-70 and S6 ribosomal protein, as shown in Fig. 7, the combinatory effect of resveratrol pretreatment and UVB irradiation reduced the activation of AKT, p-70 and S6 below that induced by UVB alone in spite of 4 hour 30′ resveratrol withdrawal. These latter results suggest that resveratrol pretreatment influence autophagy markers in UVB-irradiated HaCaT cells.

### 7. Imaging autophagosomes in vivo in response to resveratrol pretreatment and UVB irradiation

**Figure 8:**
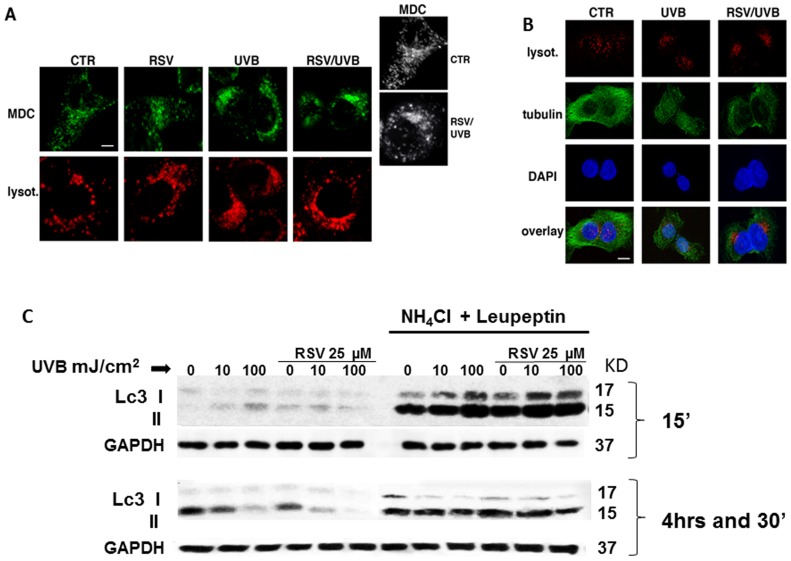
Analysis of effects of UVB irradiation on autophagosomes and lysosomes compartments and on microtubule cytoskeleton in HaCaT cells untreated or pretreated with resveratrol. (A) HaCaT cells, in control conditions (CTR) or upon different treatments (RSV, UVB, RSV/UVB) were stained with monodansylcadaverin (MDC, in green) or with lysotracker (lysot., in red) as described in materials and methods. Serial confocal sections were collected. The 3D reconstruction (black and white panels) is shown. Bar, 6 µm. (B) HaCaT cells, grown on coverslips, were loaded with lysotracker (in red), fixed and stained with a specific antibody against tubulin and revealed by FITC-conjugated secondary antibodies (green). Nuclei were labeled with DAPI (blue). Serial confocal sections were collected. Bar, 8 µm. (C) Lc3-I to Lc3-II conversion analysis in HaCaT cells exposed to resveratrol, UVB or both. HaCaT cells were pretreated for 24 hours with 25 µM resveratrol in the presence or the absence of NH_4_Cl (20 mM) and leupeptin (100 µM) and then irradiated with UVB (10, 40, and 100 mJ/cm^2^). Cell lysates were collected 15′ (panel A) or 4h30′ (panel B) after UVB irradiation and resveratrol withdrawal. A representative blot analyzing the levels of Lc3 I to Lc3 II conversion is shown. GAPDH was used as loading control.

Imaging autophagosomes *in vivo* by using the fluorescent dye monodansylcadaverine (MDC) showed a strong increase of autophagosomes in UVB-irradiated cells in comparison to control cells (Fig. 8A). Strikingly, resveratrol pretreatment does not prevent, but enhances the formation of autophagosomes (Fig. 8A), thus confirming that the combined treatment of UVB and resveratrol leads to the activation of autophagy.

Interestingly, we observed that, while in control cells autophagosomes are scattered throughout the cytosol as different-size punctate structures, both in UVB alone and in resveratrol-pretreated cells they appear as larger condensed structures (probably due to the fusion of these compartments) strictly localized in a perinuclear region (Fig. 8A). Similarly, the spatial distribution of lysosomes is altered upon these treatments (Fig. 8A). Because the dynamic positioning of cell organelles largely depends on the microtubule cytoskeleton, we investigated whether it is responsible for these alterations by staining microtubules with a specific anti-tubulin antibody (Fig. 8B). We found that, compared to control cells, UVB alone drastically led to disassembly of microtubule network (Fig. 8B). The same effect was observed in both UVB-irradiated and resveratrol pre-treated cells, although in some of them the disassembly of the microtubule network was even more pronounced in comparison to irradiated only cells (Fig. 8B), thus confirming that resveratrol enhances the effects induced by the UVB.

### 8. Combined effect of resveratrol pretreatment and UVB irradiation on the autophagic marker LC3

Finally, we analyzed LC3, microtubule-associated protein 1 light chain 3, a mammalian homolog of yeast Atg8, which is a reliable marker of autophagosomes. [41]–[44]. Tracking the conversion of LC3-I to LC3-II is indicative of autophagic activity. The amount of LC3-II increases during autophagosome formation, an initial step in autophagy, while LC3-II decreases during autophagosome-lysosome fusion and degradation of intra-autophagosomal contents by lysosomal hydrolases. LC3 conversion is a dynamic process (autophagic flux) that can be inhibited by specific inhibitors at each different steps. NH_4_Cl inhibits autophagy at a later stage by preventing the fusion of autophagosomes and lysosomes, while leupeptin, by inhibiting lysosomal cysteine protease, suppresses subsequent digestion of the autophagolysosome contents, thus stabilizing LC3-II. Thus, autophagic flux can be assessed by measuring the accumulation of LC3-II (14 kDa band) induced by the addition of inhibitors [45].

We tested the conjoint effect of resveratrol pretreatment and UVB irradiation on LC3 conversion in HaCaT cells. UVB induced LC3 conversion already 15 minutes after irradiation (Fig. 8C). Resveratrol pretreatment alone increased LC3-II, but apparently induced a decrease of the lipidated molecule in the irradiated cells. This effect of resveratrol lasted up to 4 hour and 30 minutes in spite of resveratrol withdrawal at time of irradiation (Fig. 8C). To assess whether LC3-II reduction was due to a reduced autophagic sequestration or to an increased autophagic degradation, we monitored LC3 lipidation in the presence or absence of specific inhibitors. NH_4_Cl (20 mM) and leupeptin (100 µM) totally blocked LC3-II degradation, thus supporting the hypothesis that resveratrol not only induces autophagy, but in combination with UVB accelerates LC3-II degradation into autolysosomes.

### 9. Relative contribution of apoptosis and autophagy to resveratrol enhanced UVB-induced HaCaT cell death

**Figure 9:**
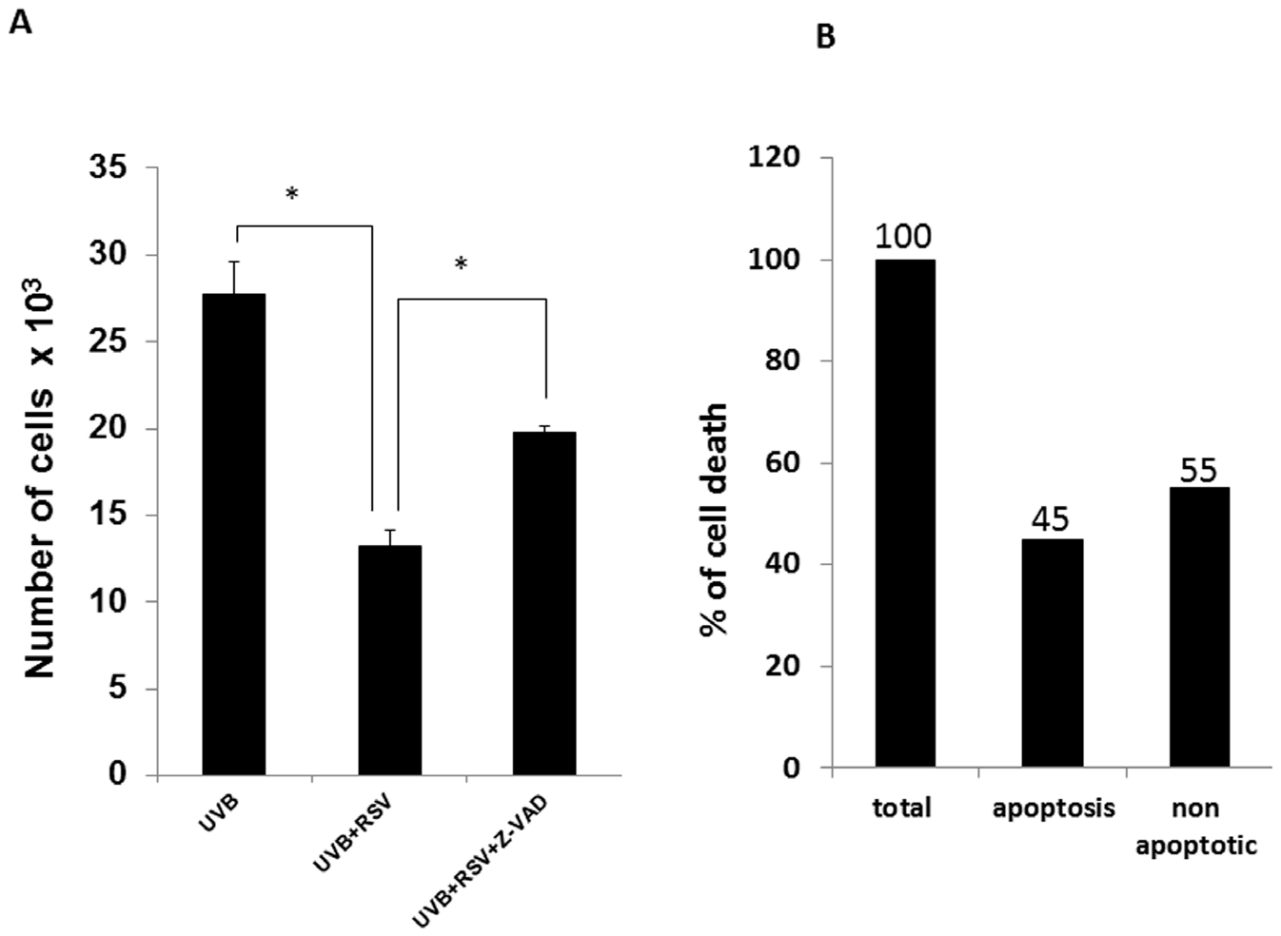
Relative contribution of apoptosis and autophagy to resveratrol enhanced UVB-induced HaCaT cells death. (A) HaCaT cells were pretreated for 24 hours with 25 µM resveratrol followed by 2 hours in the presence or absence of pan caspase inhibitor Z-VAD (50 µM) prior to irradiation with UVB (30 mJ/cm^2^). Cell count was performed after additional 24 hours of culture in standard medium. Graphs report mean values ± standard deviation of three independent measurements for each experimental point. RSV: resveratrol; * p<0.05. (B) “Total” represents the difference of cell number between UVB-irradiated HaCaT cells and resveratrol pre-treated and UVB-irradiated HaCaT cells; “Apoptotic” represent the difference of cell number between resveratrol/Z-VAD pre-treated/UVB-irradiated HaCaT cells and resveratrol pre-treated and UVB-irradiated HaCaT cells; Both “Total” and “Apoptotic” have been reported as percentage of “Total”(100% and 45% respectively). “Non-apoptotic” (55%) represent the difference of the percentages between “Total” and “Apoptotic”.

To test the relative contribution of apoptosis and autophagy to resveratrol enhanced UVB-induced HaCaT cells death, 50 µM pan caspase inhibitor Z-VAD was added to resveratrol pretreated HaCaT cell 2 hours prior to UVB irradiation. Z-VAD pretreatment, i.e. caspase cleavage inhibition, only partially affected the death enhancing effect of resveratrol pretreatment on UVB-induced HaCaT cell death as shown in Fig. 9 although totally reversing the detrimental effect of UVB alone (data not shown). Thus, all together our data strongly indicate that resveratrol enhances UVB-induced HaCaT cells death via both apoptosis and autophagy. In particular, while apoptosis accounts for 45% to cell death, autophagy contributes to the remaining 55%.

## Discussion

Resveratrol, a natural dietary polyphenol, is considered a potential candidate to modify cellular response to UVB irradiation because of its effects on redox balance [15]. In experimental settings, however, resveratrol exerts pleiotropic activities, that are highly variable depending on cell type, timing, and dosage of the compound [15], [46]–[48]; hence, it is conceivable that combining resveratrol treatment with UVB exposure might enhance the level of complexity in terms of cellular responses further beyond ROS regulation. With this in mind, in the present work we aimed at depicting the cellular and molecular response of cultured human keratinocytes, HaCaT cells, to resveratrol exposure, UVB irradiation, and both factors.

We have previously reported that in HaCaT cells resveratrol induces reversible growth arrest not associated with traits of apoptosis [39]. However, the present evidence demonstrates that in HaCaT cells resveratrol pretreatment, although reducing UVB-induced ROS production, potentiated UVB-induced apoptosis and activated alternative pathways such as autophagy, thus leading to cell death. Since, the resveratrol enhancing UVB death effect is in seeming contrast with the ROS-reducing capacity of the polyphenol we tested the effect of H_2_O_2_ in HaCaT cells pretreated with resveratrol in order to understand whether this effect was specific to UVB, or was common to other oxidant treatments. Interestingly, the levels of ROS elicited by H_2_O_2_ treatment were markedly lower than those elicited by UVB irradiation, but also in this case resveratrol-induced ROS reduction, was accompanied by increased cell death. Therefore these results suggest that the death-enhancing effect of resveratrol was independent from the type of oxidative treatment and from levels of ROS and that resveratrol-induced ROS variations are not involved in this process. In addition, in UVB irradiated HaCaT cells resveratrol pretreatment induced the quick and sustained activation of ERK and P38, both considered part of the apoptosis triggering machine. Moreover, already at 15′ after irradiation, the 24-hour pretreatment with 25 µM resveratrol increased BAX/BCL2 ratio. Taken together, the present results suggest that resveratrol pretreatment induces a precondition that irreversibly enhances the detrimental effect of UVB and H_2_O_2_. The observation that, 4 hours and 30′ after resveratrol withdrawal and UVB irradiation, UVB-induced cleavage of both caspase 8 and PARP was enhanced in resveratrol pretreated HaCaT cell and that in addition Bax/Bcl2 ratio remains elevated provide the molecular basis to support this hypothesis. This should be not surprising since, in various cancer types, resveratrol behaves as a chemosensitizer that lowers the threshold of cell death induction by classical anticancer agents, thus counteracting tumor cell chemoresistance [49]–[53].

In this light, induction of traits autophagy such as reduction of AKT, m-TOR and S6 activation caused by resveratrol pretreatment of in UVB irradiated HaCaT cells open an other trail through which resveratrol can exert a protective effect in keratinocytes. It is also important to notice that the reversible effect of resveratrol alone on autophagic markers is in contrast with the long lasting effect of resveratrol pretreatment in combination with UVB irradiation. In fact, in spite of a significant recovery of the AKT-m-TOR pathway activation detectable 4 hours and 30 minutes since withdrawal of the polyphenol, in UVB-irradiated HaCaT cells pretreated with resveratrol significant inhibition of the m-TOR pathway was still observable 4 hours and 30 minutes after resveratrol removal. These different responses could reflect the reversible and irreversible effects of resveratrol depending on the context. Therefore, in the samples pretreated with resveratrol alone, the strong signals of phospo-AKT and phospho-S6 observable 4 hours and 30′ after resveratrol withdrawal are related to the cessation of the growth inhibitory effect of resveratrol, the recovery of cell proliferation and the possibly associated rebound effect on the activation of the two analyzed markers. Viceversa, upon UVB irradiation resveratrol-induced modification became irreversible as demonstrated by the sustained inhibition of the levels of AKT and S6 phosphorylation.

Moreover, we have reported a sustained activation of ERK 1/2, in line with the suggested autophagy induction via ERK 1/2 [54], [55]. In a recent study, this Erk1/2-dependent autophagy activation has been shown to be controlled by m-TOR, which affects Beclin1, the central autophagy protein with an important role in the cross-talk with the apoptosis pathway [56]–[57]. To try to shade light on the respective contribution of apoptosis and autophagy in cell death we have tested the effect of resveratrol pre-treatment on the expression of Beclin1. The observation that resveratrol alone induces reversible growth arrest and Beclin1 expression suggests that in HaCaT cells resveratrol induces a canonical pro-survival autophagy. On the other hand, in irradiated cells pre-treated with 25 µM resveratrol both at 2 and 24 hours, beclin1 expression inversely correlates with caspase 8 and PARP cleavage, while pre-treatment with 100 µM induced beclin1 expression only at 2 hours which progressively decreased at increasing doses of UVB. Thus, one can speculate that, upon resveratrol pre-treatment prior to UVB irradiation, three different scenarios are possible: at the higher doses of UVB, the majority of cells will be substantially damaged and resveratrol pretreatment with both at high and low concentration of resveratrol will enhance apoptosis regardless the time of resveratrol pre-treatment; at low dose of UVB cells will be moderately damaged and low and high concentrations of resveratrol will induce autophagy, possibly with survival purpose; however, because the UVB-induced damages, autophagy will be massive, thus leading to cell death; finally, because UVB-induced damages are stochastic events, then, within the same cell population, cells with various degree of damage co-exist and die according to their different status either of apoptosis or massive autophagy. Whatever the scenario, resveratrol-induced autophagy in UVB-irradiated HaCaT cells cannot be considered as a cellular adaptation leading to survival, but rather as a damaged cell response leading to death. This is also confirmed by autophagosomes increase upon combined treatment of resveratrol and UVB, which in turn induce a drastic alteration in the structure and spatial distribution of both autophagosomes and lysosomes and in the organization of the microtubule network. Autophagy is a dynamic process referred to as autophagic flux that is responsible for autophagosome synthesis, delivery of autophagic substrates to the lysosomes, and degradation of autophagic substrates inside lysosomes. In the process of autophagosome synthesis, the cytoplasmic microtubule-associated protein 1 light chain 3-I (LC3-I) is conjugated with phosphatidylethanolamine (PE) and becomes the membrane-bound form LC3-II, initially located in both the outer and inner membranes of the autophagosomes. LC3-II is then removed from the outer autophagosomal membrane after the fusion between autophagosomes and lysosomes or late endosomes [41], [42] where LC3-I and -II are completely degraded. In HaCaT cells resveratrol induces LC3-I to LC3-II conversion and increases that induced by UVB. Disappearance of both LC3-I and -II in cells treated with increasing doses of UVB and resveratrol suggests that the combined effect of UVB and resveratrol increases or speeds up the fusion autophagosomes –lysosomes and consequently the degradation of LC3. Experiments with NH_4_Cl and leupeptin, which by different means interfere with lysosome activity, totally block LC3-II degradation, thus strengthening the hypothesis that resveratrol does interfere with autophagic flux in this system by increasing LC3 degradation.

The molecular mechanisms coordinating autophagy and apoptosis are not fully established. Apoptosis and autophagic cell death may exist as cooperative or competitive pathways depending upon the cellular environment. For example, caspase inhibitors can prevent apoptosis and promote other forms of cellular death, such as autophagy. Stimulation of both apoptotic and non-apoptotic cell death can also occur in some circumstances [54]. Several anti-neoplastic drugs can induce not only apoptosis, but also autophagic cell death, in overlapping or parallel scenarios [58]. In the present work, we provide evidence of the coexistence of enhanced apoptosis and autophagy in human keratinocytes when UVB irradiation is preceded by treatment with resveratrol. We think that the relevance of autophagy in the present system, where cells are undergoing also apoptosis, consists in providing an additive effect that drives a larger fraction of UVB-irradiated cells to death. This might be biologically relevant also in *in vivo* settings, reducing the odds that UVB-transformed cells might escape death and undergo malignant proliferation.

Resveratrol holds great potential not only in the prevention, but also in the therapy, of skin diseases because of its ability to interfere with multiple cellular pathways whose regulation profoundly differ in normal and cancer cells. In this view the ability of resveratrol to enhance death of the non-tumorigenic HaCaT cells following UVB irradiation, is potentially relevant to different hyperproliferative skin diseases. In that, to better exploit resveratrol repertoire in prevention and therapy it is not only important what resveratrol is able to do by itself, but also what it is able to generate after interacting with other factors like UVB.

In conclusion, our observations support the idea that resveratrol endorses a “zero-tolerance” cellular response to UVB induced damages, thus removing all sort of damaged cells that could turn out to be transformed in the long run. Finally, it is tempting to speculate that the association of apoptosis with autophagy [59], [60] could provide a mechanistic rationale for coupling the anticancer with the longevity potential of resveratrol.
